# Structural DNMT-nucleosome contacts are related to DNA methylation patterns

**DOI:** 10.1186/s13072-025-00626-1

**Published:** 2025-09-12

**Authors:** Kevin George, Kerstin Neininger, Anna Elizabeth Schmitz, Jörn Walter, Volkhard Helms

**Affiliations:** 1 Center for Bioinformatics, Saarland University, Saarbrücken, Germany; 2 Department of Genetics, Saarland University, Saarbrücken, Germany

**Keywords:** Steric accessibility, Structural superimposition, DNMT, NOMe-seq

## Abstract

DNA-methylation is a key epigenetic mark in chromatin that attenuates chromatin accessibility during transcription, implying a crucial role in gene regulation. Its symmetrical distribution and function is thought to be linked to the periodicity of the DNA helix and the positioning of DNA wrapped around the nucleosome. Epigenomic data suggest that DNA methyltransferases (DNMTs) can methylate DNA when wrapped around a histone octamer. Yet, how this is precisely linked to positioning and periodicity is yet to be elucidated. It has been hypothesized that the observed methylation patterns may be related to the changing accessibility of nucleosome-bound DNA to DNMTs. Here, incorporating NOMe-Seq data, which simultaneously measures nucleosome positioning and DNA methylation at CpG sites across the genome, the interaction of DNMT1 with nucleosomal DNA could be mechanistically modeled and compared to hypothesized dependencies. Furthermore, X-ray structures of DNMT1 were superimposed onto those of nucleosome core complexes at base resolution to determine which histone-bound DNA positions would be sterically accessible or inaccessible to DNMTs. Statistical comparison with experimental NOMe-Seq data revealed that structurally computed DNA accessibility scores can indeed explain DNA methylation patterns in actively transcribed regions with positioned high nucleosome density.

**Supplementary Information:**

The online version contains supplementary material available at 10.1186/s13072-025-00626-1.

## Introduction

Eukaryotic DNA is hierarchically organized into chromatin, with the nucleosome representing the smallest repeating unit [1, 2]. The nucleosome core consists of two copies of a set of four histone proteins (H3, H4, H2A, H2B), with approximately 145–147 bp of double-stranded DNA wrapped around the histone octamer [1–3]. On average, the periodicity of the DNA double-helix yields approximately 10.2 bp per turn when bound to the nucleosome and around 10.5 bp per turn for free DNA [2]. Single nucleosomes are connected via linker DNA, with a length ranging from 10–80 bp [4] to form a bead-like string structure that is, in turn, packaged into folded chromatin fiber by short-range nucleosome-nucleosome contacts. Higher order chromatin structure is then formed by long-range fiber-fiber interactions, begetting the structure of a condensed chromosome [3, 5, 6].

Chromatin is highly dynamic and plays a central role in the regulation of replication, gene transcription, and DNA repair [1, 3, 7]. Open chromatin is commonly associated with transcriptionally active genes, whereas condensed chromatin is assumed to be implicated in gene silencing [4, 8]. In addition to chromatin state, further chromatin modifications, such as post-translational histone modifications, DNA methylation, and chromatin remodeling complexes, are crucial components in the regulation of gene expression [8, 9]. Moreover, evidence exists purporting that the nucleosome may also serve a role in positive gene regulation in addition to transcriptional repression [10].

In eukaryotes, DNA methylation classically refers to the addition of a methyl group to the C-5 position of a cytosine ring [11]. Although this commonly occurs in a CpG context (98%), non-CpG methylation has been detected in mammals, as well [12–14]. DNA methylation is vital for the trajectory of an organism’s development, as well as genomic imprinting [15, 16], X-chromosome inactivation [15, 17], and repression of transposable elements [18, 19]. Promoter hypermethylation is associated with cancer development, as this epigenetic modification may result in transcriptional silencing of tumor suppressor genes [20].

DNA methylation is catalyzed by DNA-methyl-transferases (DNMTs), whereby DNMT1 mainly functions in the context of DNA methylation maintenance and DNMT3a/3b carries out *de novo* DNA methylation [11, 21]. In contrast to DNMT3a/3b, DNMT1 recognizes and targets hemimethylated CpG dinucleotides [21]. DNMT1 is composed of several protein domains: of which, the CXXC domain binds to DNA possessing unmethylated CpG sites [21]. Further DNMT1 domains include a C-terminal catalytic methyltransferase domain, a bromo-adjacent homology domain (BAH1/2), and a replication foci-targeting domain (RFD) [21].

It has been previously reported in *A. thaliana* [22] and *H. sapiens* [23], that nucleosome-bound DNA tends to be more heavily methylated than the surrounding flanking DNA. Notably, the DNA methylation levels of nucleosomal DNA exhibit a ten-base periodicity. DNMTs typically bind to the major groove of DNA, which alternatively faces toward or points away from the nucleosome in accordance with the ten nucleotide periodicity of double-stranded DNA (dsDNA) [22]. The correlations between these patterns suggest that DNMTs are capable of methylating nucleosome-bound DNA. Overall, nucleosome occupancy and DNA methylation appear to be interdependent [22].

Nucleosome positioning, in tandem with DNA methylation, are essential for the regulation of gene expression [24]. NOMe-Seq (Nucleosome Occupancy and Methylome Sequencing) illuminates genome-wide nucleosome position footprints and CpG DNA methylation patterns from the same DNA molecule [24]. Nucleosome occupancy information is informed by GpC methyltransferase M.CviPI’s ability to access individual GpC sites [25]. The modified methylome can then be probed via whole-genome bisulfite sequencing (WGBS). To avoid potential cross-talk between artificial and native methylation, cytosines in GCG context are discarded. Thus, four different methylation patterns emerge: nucleosome occupied and nucleosome depleted regions, which may be classified as either methylated or unmethylated.

This study aims to determine the interplay between the nucleosome occupancy of DNA and the ability of DNMT1 to methylate CpGs at positions relative to the nucleosome. We investigated the existence of DNA positions not reachable by the DNMT1 enzyme due to organization of nucleosome structure. For this, an *in silico* structural superimposition approach was implemented and the computed accessibility scores were subsequently compared to experimental NOMe-Seq methylation data. Two nucleosome conformations were analyzed, representing a packed and unpacked state, to assess how accessibility varies with nucleosomal packing. Additionally, RNA-Seq data was employed to examine how accessibility scores align to methylation patterns in expressed and non-expressed genes. It was determined that, when DNA is wound around the histone octamer in the nucleosome complex, the DNA methylation pattern in actively transcribed regions with high nucleosome density reflects the accessibility of specific DNA positions to DNMT1.

## Methods

### Data

NOMe-Seq data was collected from the immortalized human liver carcinoma cell line HepG2, from the DEEP (German Epigenome Project) consortium (Dataset accession id: EGAD00001002527), via the European Genome-Phenome Archive. RNA-Seq data from HepG2 was obtained from NCBI (GEO accession: GSE206417) [26]. The first of two replicates was analyzed from the data.

### Processing of NOMe-Seq

#### Raw data

FASTQ files were trimmed to remove adapter sequences and low-quality sequences (Q < 20) using Trim Galore (version 0.6.10) [27]. Subsequently, the raw NOMe-Seq reads were mapped to the human reference genome GRCh38.p14 (https://www.gencodegenes.org/human/) using Bismark (version 0.24.2) [28] in conjunction with Bowtie 2 (version 2.5.1) [29]. Next, the Bis-SNP pipeline (version 0.82.2) [30] was implemented to extract methylation levels for cytosines in both a GCH and HCG context, H signifying the IUPAC code for A, C, or T. This process resulted in the output of two BED files, one for each context, including information for each GpC or CpG site. For further analysis, the BED files were filtered to include only sites with a read coverage greater than 3.

#### LNDR and HNDR detection

The resulting GpC methylation data were utilized to identify low nucleosome dense regions (LNDRs). Then, methylated and unmethylated C’s were counted at each GCH site in 200-bp windows with 20-bp steps, followed by the application of a $\chi ^2$ test to compare against the genome background. A *p*-value cutoff of $10^{-5}$ was established to determine windows displaying statistical significance. Overlapping windows (adjacent or $\ge$ 1-bp overlap) were merged to resolve the final LNDR regions [31], which may likely indicate open chromatin conformation. Genomic regions complementary to LNDRs, possessing higher nucleosome density relative to their surroundings (HNDRs), were identified using the *complement* sub-command of the BEDTools suite (version 2.27.1) [32].

**Fig. 1:**
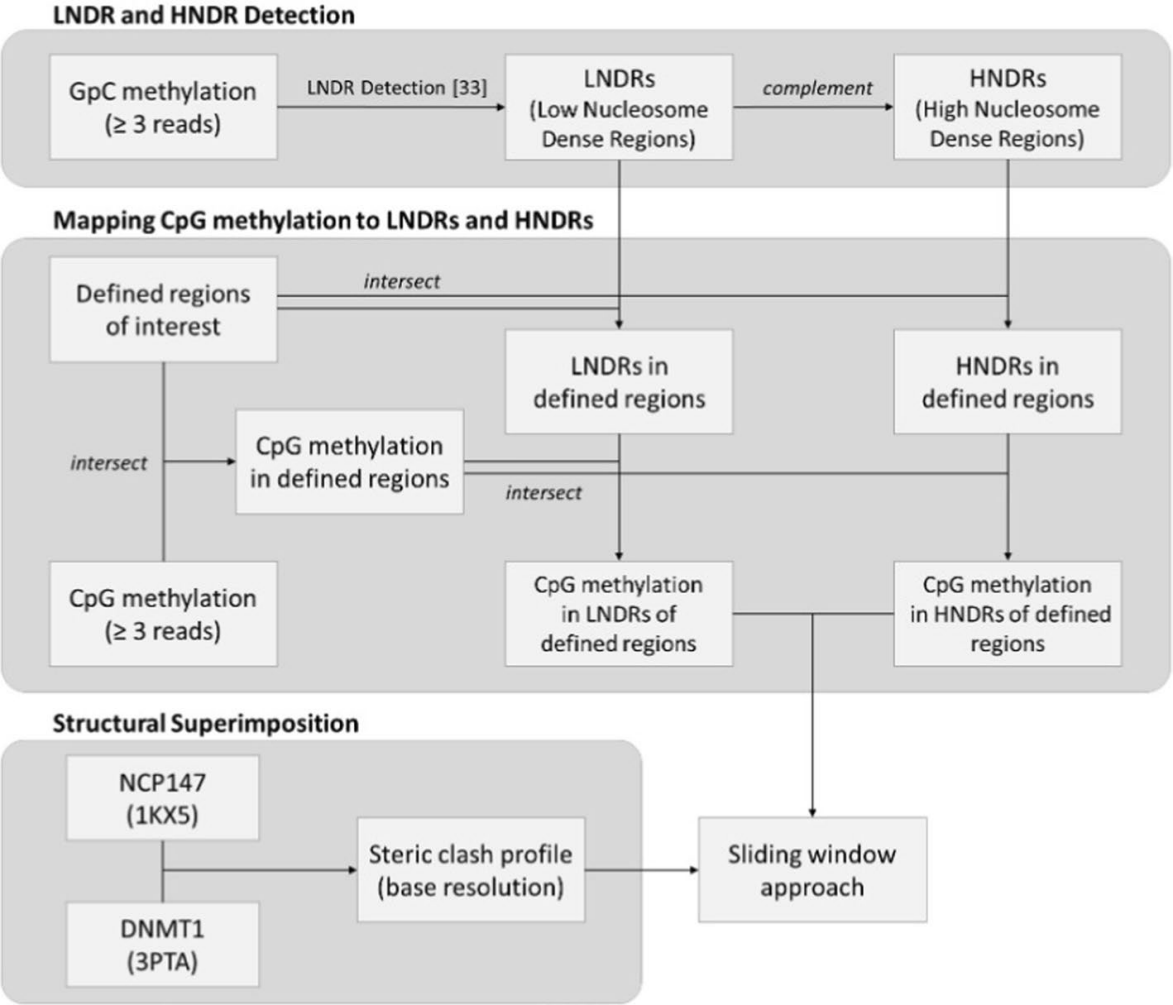
Workflow for comparing *in silico* and experimental data. The flowchart illustrates how accessibility values (steric clash profile) obtained via *in silico* structural superimposition were compared to experimentally observed methylation levels. Analysis was carried out separately for expressed and non-expressed genomic regions

### Comparison with experimental methylation data

#### Regions in study

The estimated DNA accessibility results were compared with experimental human gene methylation data in four specific genomic regions of interest: the promoters, the start of the $1^{\textrm{st}}$ intron, the end of the $1^{\textrm{st}}$ intron, and the beginning of the 2^nd^ intron. Promoter regions are known to harbor specific nucleosome patterns that enable transcriptional regulation [24]; in order to examine these patterns within the data, promoters were defined within the range of – 2000 to + 1000 bp around the transcriptional start site (TSS). Similarly, studies suggest that exon-intron boundaries also exhibit nucleosome phasing [33]. Analogous to the promoters, the same regions from – 2000 to + 1000 bp relative to the start of the 1^st^ intron, the end of the 1^st^ intron and the beginning of the 2^nd^ intron were considered.

#### CpG methylation levels in LNDRs and HNDRs

Resulting CpG methylation was mapped to the detected LNDRs and HNDRs for all four defined genomic regions. To this aim, the LNDRs and HNDRs were initially mapped to each defined region using the *intersect* function of the BEDTools suite. The *intersect* function was utilized once more to overlay the CpG methylation data onto the LNDRs and HNDRs of each region, with the full workflow of which illustrated in Fig. 1.

#### Structural superimposition approach

**Fig. 2:**
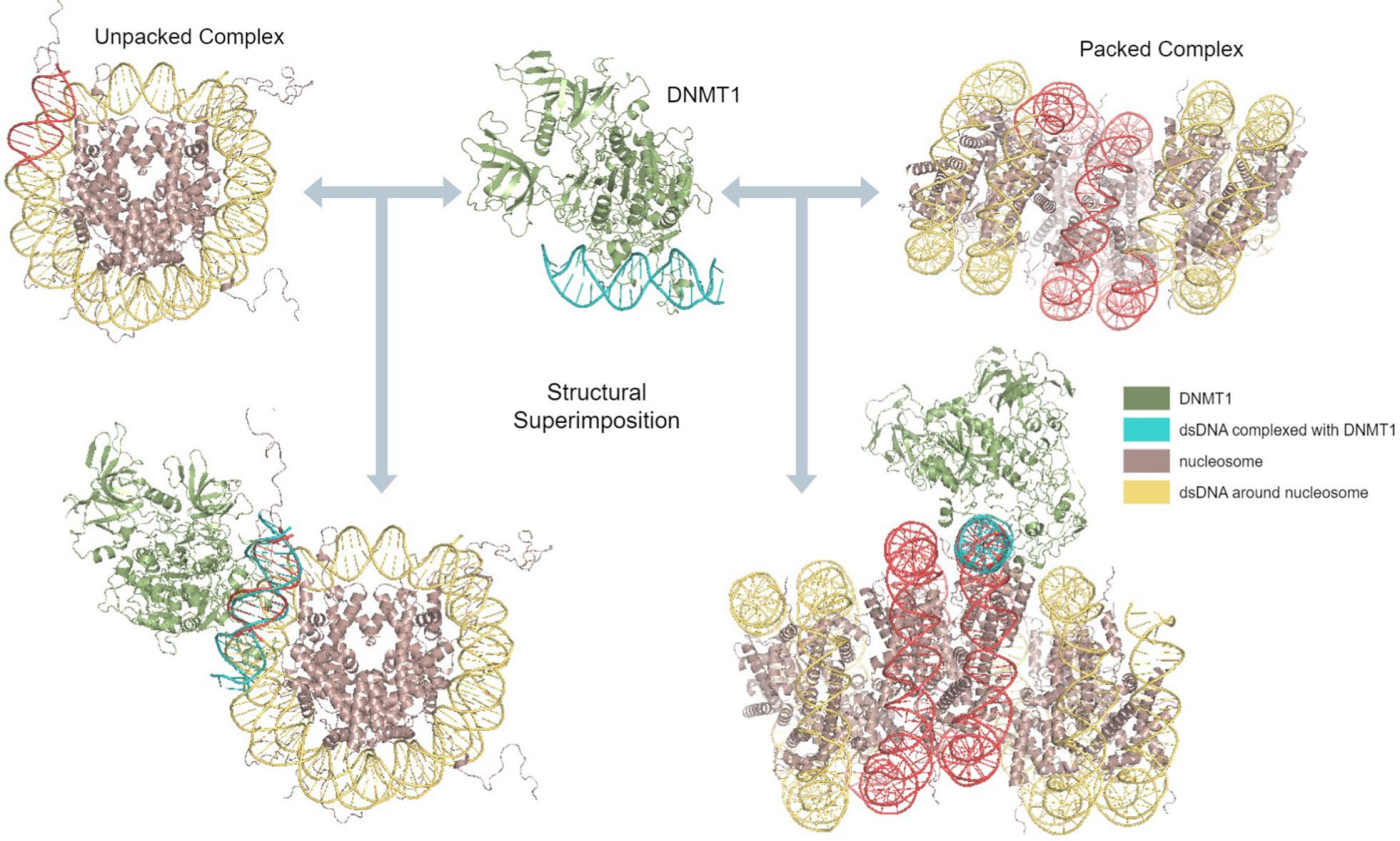
Structural superimposition approach: initially, DNMT1 is positioned relative to the nucleosome complex by mapping DNA base pairs 4–15 of DNMT1 onto different positions of the nucleosomal DNA; then, the respective rotation and translation matrices are applied to the spatial coordinates of DNMT1. For the unpacked nucleosome, DNMT1 was mapped onto all possible positions along the 147 base pairs of DNA wound around the complex. The figure illustrates DNMT1-bound DNA aligned to the nucleosome DNA at position 1. For the packed nucleosome, DNMT1 was mapped to the 147 base pairs of DNA wound around the central nucleosome, as highlighted in red. In both cases, DNMT1 is oriented away from the nucleosome complex, which appears to represent a plausible spatial arrangement

First, a structure of human DNMT1 bound to a 19 bp DNA molecule (PDB ID: 3PTA; residues 646-1600) was retrieved from the Protein Data Bank (PDB) [34]. Additionally, we considered a cryoEM structure of human DNMT1 in an active conformation bound to 12 bp hemimethylated DNA, displaying a flipped out 5fC base (PDB ID: 7XIB; residues 264-1259) [35]. Two models were considered to visualize the manner in which DNA packs around nucleosomes: a single nucleosome core particle (PDB ID: 1KX5) representing an unpacked nucleosome complex, and a telomeric trinucleosome complex (PDB ID: 7V9J) representing a packed nucleosomal complex. Biopython [36] was utilized to perform structural superimposition of DNMT1 onto each alternative histone-bound DNA position for both nucleosome complexes, as displayed in Fig. 2.

To create the structural alignment, the atomic positions of the DNA deoxyribose sugar backbone in the DNMT1-DNA complex were aligned with those in the nucleosomal DNA. For the DNMT1-DNA complex, DNA base pairs 4 to 15, 12 base pairs in total, were used for alignment. In the unpacked nucleosomal complex, all 147–11 base pairs were considered as putative alternative alignment positions for the 12 base pair stretch, resulting in 136 unique structural alignments. Similarly, for the packed nucleosomal complex, DNA base pairs 127 to 273, 147 base pairs total, were used to focus on the DNA surrounding the central nucleosome, also resulting in 136 unique alignments. For each possible structural alignment between DNMT1-DNA and nucleosome-bound DNA, Biopython was used to perform a superimposition that minimized the root-mean-square deviation (RMSD). The rotational and translational matrix ($M_{RT}$) needed to optimally align the sugar backbone of the DNMT1-bound DNA with the nucleosome-bound DNA was subsequently calculated. The same $M_{RT}$ was then applied to all atoms of the DNMT1 molecular complex, resulting in a spatial transformation aligned with the specific nucleosome-DNA position.

Next, the mechanistic feasibility of each DNMT1-nucleosome superimposition was assessed by evaluating the extent of steric clashes between DNMT1 and the nucleosome at each binding position. It is assumed that a steric clash exists between two atoms if $d<r1+r2$, with *r*1 and *r*2 defining the respective atomic van der Waals (vdW) radii and *d* signifying the distance between the two atoms. To quantify the overall steric clash  expressed as a percentage [%] between the DNMT1 enzyme and the nucleosome at a given DNA position, the number of steric clashes were counted between DNMT1 and the nucleosome atoms, excluding DNA atoms, and normalized by the total number of DNMT1 atoms.

#### Sliding window approach

**Fig. 3:**
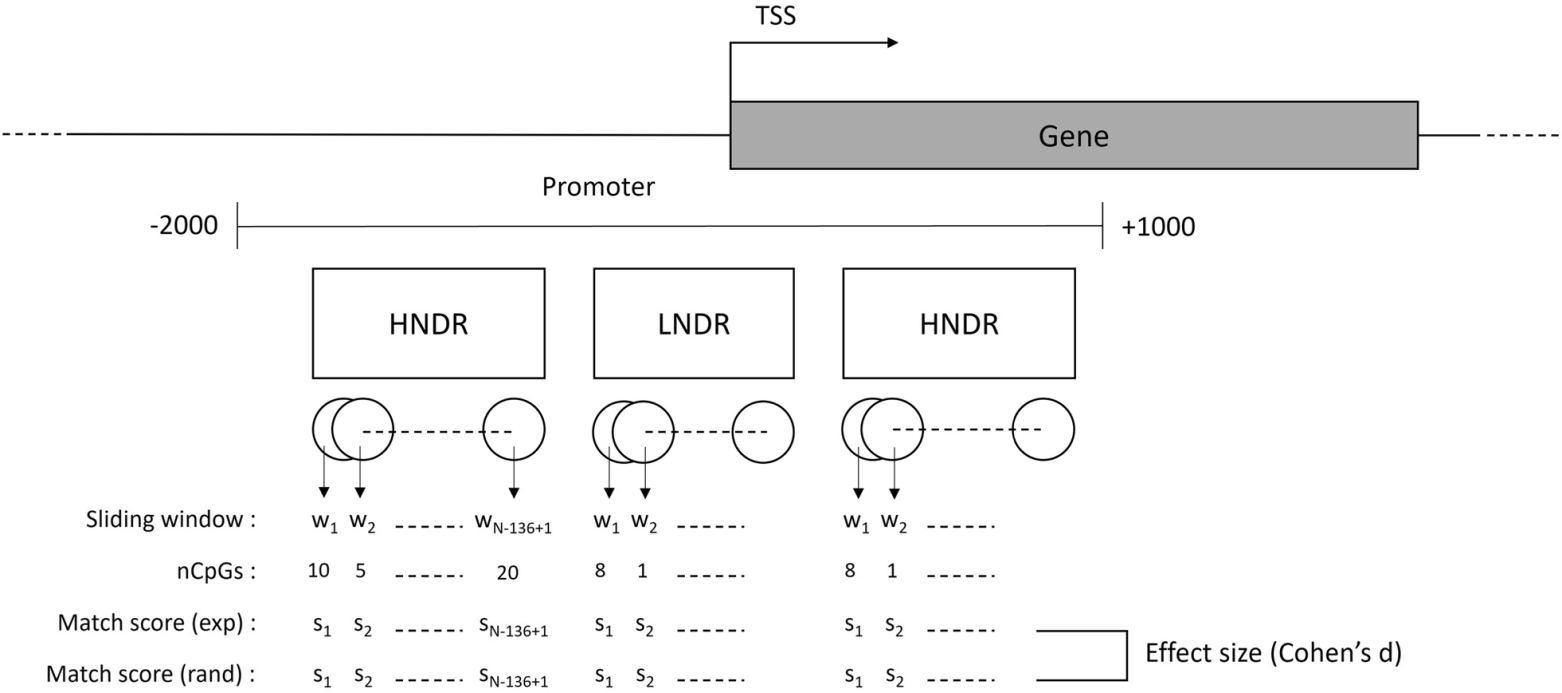
Sliding window approach: shown is a promoter region, defined to range from – 2000 to + 1000 bp relative to the TSS. Matching–scores were calculated for all possible sliding positions within HNDRs and LNDRs. To evaluate the results, scores between experimental and randomized data were subsequently compared with respect to Cohen’s d values and *p*–values calculated with the Wilcoxon rank–sum test, in order to measure effect size and statistical significance, respectively

To quantify the correspondence between *in silico* accessibility values and DNA methylation frequencies, a sliding window approach was applied, calculating matching scores for each possible window position. The window length was set to 136 bp, corresponding to the number of nucleosome positions with clashing information, while the step size was set to 1 bp, as outlined in Fig. 3.

For all defined regions with HNDRs and LNDRs, a $match-score \in [0,1]$ was calculated for each sliding window position *w* as:

$$\begin{aligned} match-score_w = \frac{1}{nCpGs_w} \sum \limits _{i=1}^{nCpGs_w}M_i \end{aligned}$$

where $M_i$ is defined as:

$$\begin{aligned} M_i = {\left\{ \begin{array}{ll} 1, & \begin{aligned} & (m_i>m_{thres} \, \text {and} \, c_i\le c_{thres}) \, \text {or} \\ & (m_i\le m_{thresh} \, \text {and} \, c_i > c_{thres}) \end{aligned} \\ 0, & \text {otherwise.} \end{array}\right. } \end{aligned}$$

Here, $m_i$ is the methylation level at CpG position *i* (%), $m_{thres}$ is the thresholding methylation level above which a CpG was considered as methylated (%), $c_i$ is the accessibility, determined by steric clash, at position *i* calculated by the structural approach (%), and $c_{thres}$ is the tolerated steric clash (%). Thus, $M_i=1$ if a CpG is methylated and the steric clash is tolerated, signifying that DNMT1 is able to bind; else, $M_i=0$ if a CpG is not methylated and the steric clash is not tolerated, indicating that DNMT1 is not able to bind. The sum was normalized by the number of CpGs in the considered sliding window. Higher match-scores suggest that the methylation level corresponds with the calculated steric clash. Match-scores were computed using a set of values for the thresholding parameters: $c_{thres} \in [5,10,20,50]$ and $m_{thres} \in [0,10,20]$, abbreviated as a combination of $c_{thres}$ and $m_{thres}$. For instance, c5m0 denotes that the score was calculated using $c_{thres}=5$ and $m_{thres}=0$, whereby steric clashes are tolerated if $c_i < 5\%$ and a CpG is assumed to be methylated if the methylation level $m_i>0$.

Next, the matching scores of HNDRs and LNDRs were compared with those derived from randomly shuffled data using the Wilcoxon rank-sum, which assesses the differences between the match scores of experimental and randomly shuffled methylation levels. However, with large sample sizes, *p*-values may become skewed to suggest higher significance, including in cases where observed difference between groups is minimal [37, 38]. In order to further assess the magnitude of these differences, the effect size (ES) was calculated using Cohen’s d, an effect size estimator independent of sample size [37, 39], as defined by:

$$\begin{aligned} d = \frac{(m_e - m_r)}{\sqrt{\frac{s^2_e + s^2_r}{2}}} \end{aligned}$$

such that $m_e$, $m_r$, $s_e$, and $s_r$ are the means and standard deviations of experimental and randomized data, respectively. Effect sizes for the difference between independent means were classified as small ($0.2 \le d < 0.5$), medium ($0.5 \le d < 0.8$), or large ($d \ge 0.8$) [37, 40].

#### Comparison on expressed and non-expressed genes

To evaluate the alignment between structural accessibility and DNA methylation in expressed and non-expressed genes, genes were categorized on the basis of expression levels, such that genes with Transcript Per Million (TPM) values greater than zero were classified as expressed, while those less than or equal to zero were considered non-expressed. To proceed, a sliding window approach was applied, as described previously, to the HNDRs and LNDRs of both gene groups to calculate the match score. Finally, Cohen’s d was computed across the dataset to evaluate the effect size relative to randomly shuffled data, for both expressed and non-expressed genes.

## Results and discussion

### Nucleosome occupancy and CpG methylation

Conducting analysis across chromosomes 1–22, X, and Y, 106,261 promoter regions, 104,611 1^st^ introns (including both start and end positions), and 98,596 2^nd^ introns were identified based on annotations from the human reference genome, GRCh38.p14. Upon processing, cytosines in both GpC and CpG contexts with less than 5 reads of coverage were excluded. The following analysis focuses on transcribed genomic regions and studies how nucleosome architecture and DNA accessibility are connected. Corresponding analyses of non-transcribed regions are presented in the supplemental material and are discussed concisely in the main text.

**Fig. 4:**
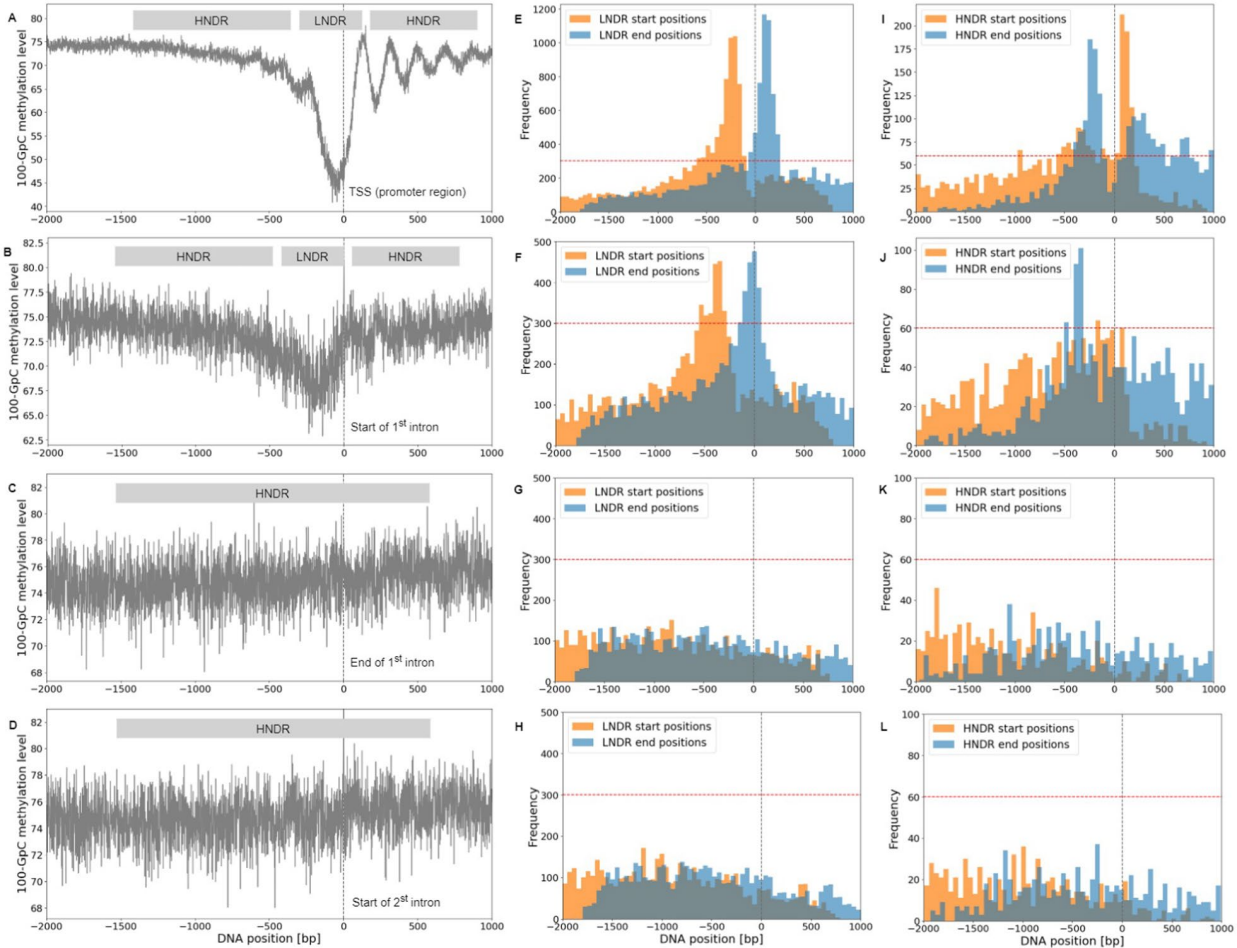
To the left, **A**–**D** display NOMe–Seq GpC patterns of expressed genes in each of the four regions of interest as average 100–[GpC methylation] levels (percent of unmethylated GpCs). Regions featuring higher nucleosome density than local surroundings (HNDRs) and regions with lower nucleosome density (LNDRs) were derived from the experimental GCH NOMe–Seq data. Middle panels E-H show start/end distributions of respective LNDRs, while to the right, **I**–**L** display such values for HNDRs

GpC sites of sufficient read coverage were mapped to four defined genomic regions in order to visualize global nucleosome patterns, as shown in Fig. 4A–D. An elevated 100–[GpC methylation] level corresponds to nucleosome-protected DNA sequences, whereas lower levels indicate a lesser degree of protection. In promoters of transcribed genes, there exists a visible decline in the 100-[GpC methylation] level immediately before the TSS, suggesting a presence of LNDRs. Additionally, a clear nucleosome phasing pattern emerges immediately downstream of the TSS in the promoter region. A similar, yet less defined pattern is observed around the start of the 1^st^ intron, featuring an observable decline around the – 200 bp mark before the start of the 1^st^ intron, with the nucleosome phasing becoming apparent thereafter. Due to the differing dimensions of LNDR stretches between genes, the upstream regions in the 1^st^ intron and promoter exhibit higher average "disorder", resulting in nebulous, less discernable nucleosome phasing, as corroborated by previous results [24]. In contrast, regions at the end of the 1^st^ intron and the start of the 2^nd^ intron reveal a consistent 100-[GpC methylation] level, indicating a general absence of LNDRs in these areas. For comparison, around the promoters of non-expressed genes, 1-GpC methylation levels drop only by approximately 7%, as opposed to 30% for expressed genes; see Figure S1. However, no pattern is visible for the start of the 1^st^ intron.

Figures 4E–H and I–L illustrate distributions of start and end positions from detected HNDRs and LNDRs across all four regions of interest. In the promoter region, most LNDRs arise approximately 200 bp upstream of the TSS and extend to around 100 bp downstream of the TSS, with HNDRs located in complementary positions. This finding aligns with the implications of Fig. 4A and precedent findings [24], supporting the accurate annotation of HNDRs and LNDRs from NOMe-Seq data. A similar pattern is observed around the start of the 1^st^ intron, where most LNDRs originate approximately 400 bp upstream and end at the start of the intron. However, no distinct patterns emerge at the end of the 1^st^ intron and at the start of the 2^nd^ intron, which proves consistent with corresponding average GpC methylation levels shown in Figs. 4C and D.

**Fig. 5:**
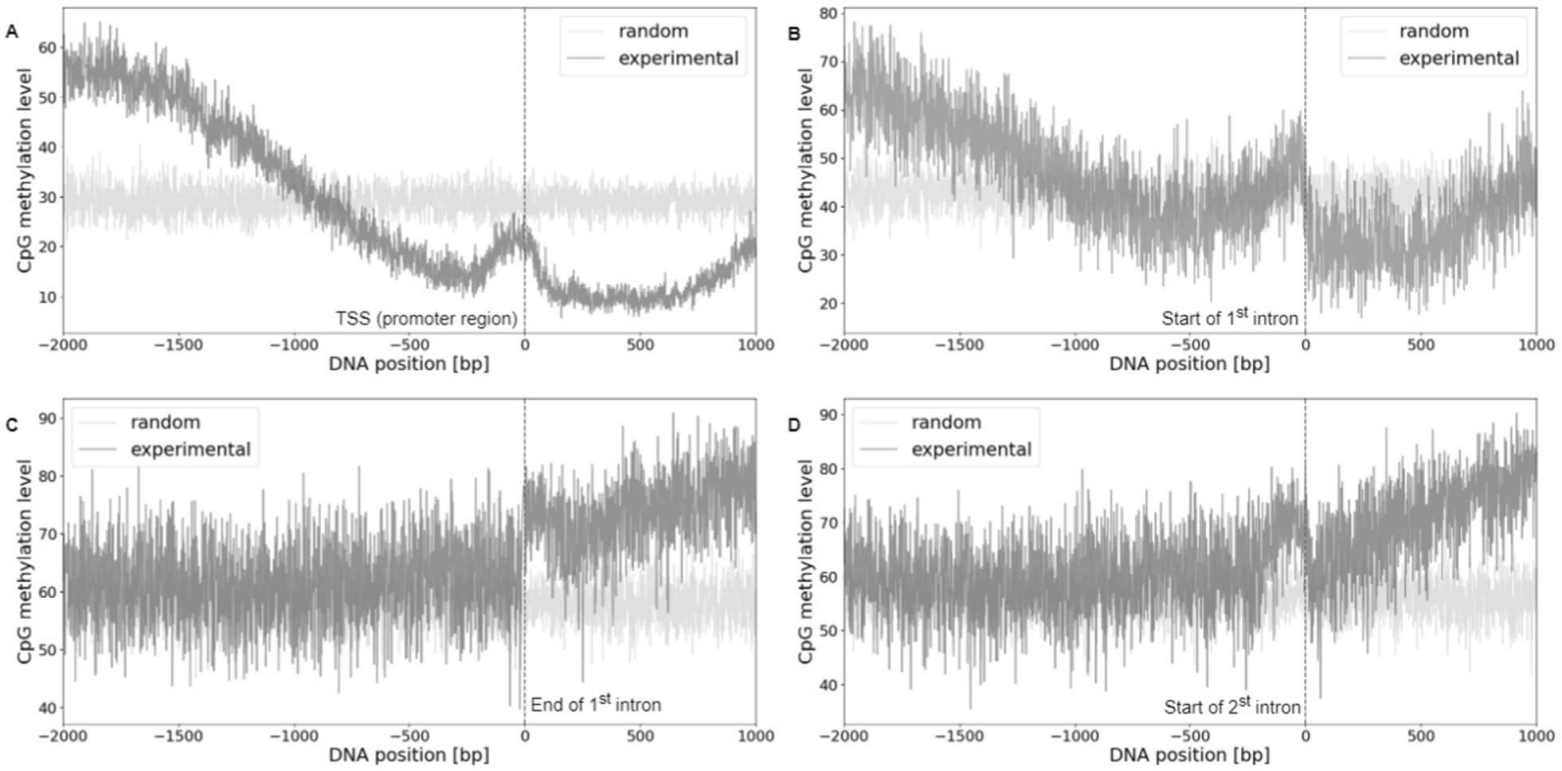
CpG methylation pattern for expressed genes in each of the four regions of interest. Experimentally observed CpG patterns (dark grey) are distinct from randomized methylation levels (light grey)

In Fig. 5, the average CpG methylation levels of expressed genes across all four regions can be compared. Methylated CpG positions were identified by assigning the filtered CpG sites to the specified regions, as shown in Fig. 1. In both the promoter regions and the start of the 1^st^ intron, CpG methylation levels decrease as they approach the relative zero position. This reduction is more pronounced for the promoter, from 55% to 15%, than for the start of the 1^st^ intron, from 65% to around 40%. Following this decline towards the TSS, the plot exhibits a small peak at the zero, proceeded by a downstream increase. However, a distinct methylation pattern hallmarks the end of the 1^st^ intron and the start of the 2nd intron. Here, CpG methylation remains constant as it approaches the relative zero position, proceeded by a small jump at the zero position, followed by a slight increase. For comparison, around the promoters of non-expressed genes, CpG methylation levels drop nominally, from around 35% to 25% for the promoters of non-expressed genes, and by approximately 5% for the start of the 1^st^ intron; see figure S2. No pattern is visible for the other two regions of interest.

### Structural superimposition

**Fig. 6:**
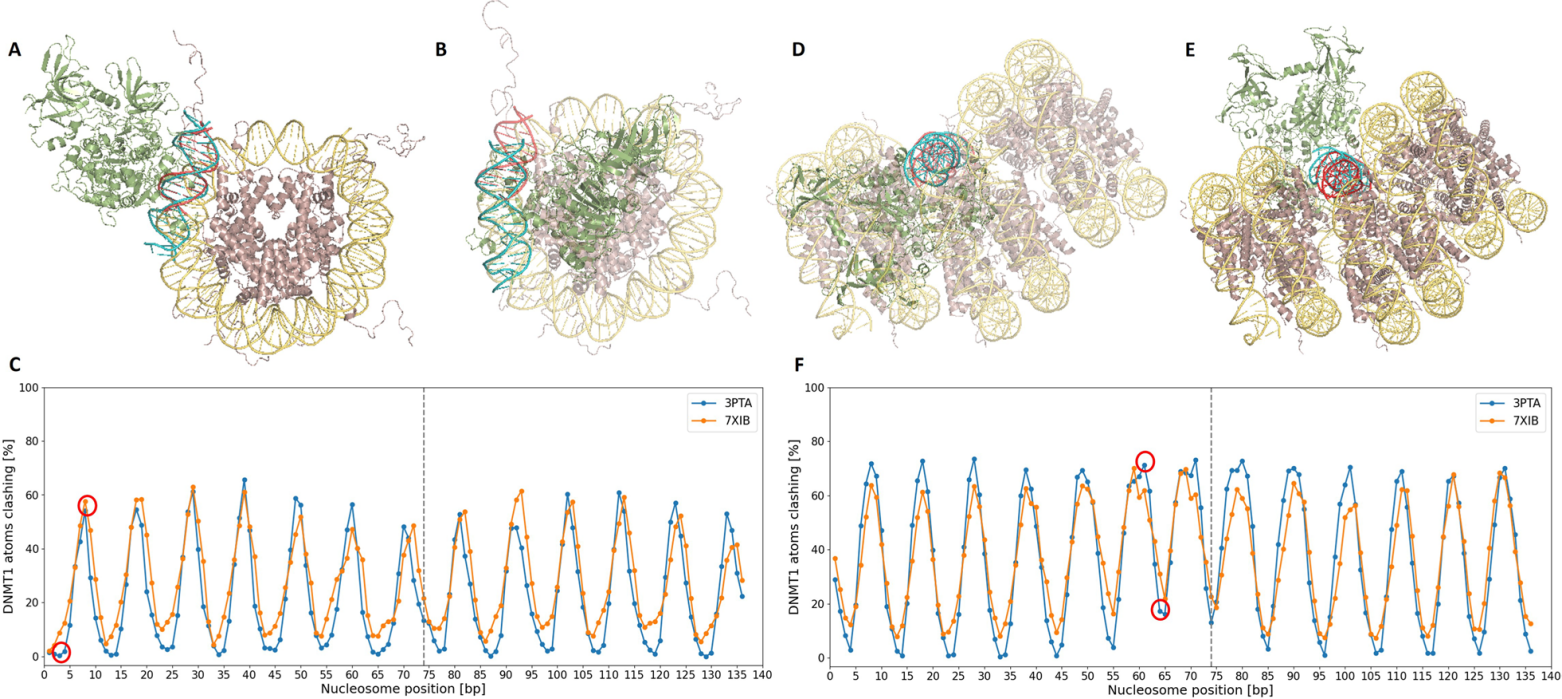
Estimated degree of steric clashing between DNMT1 and the nucleosome core complex. The top panel depicts superimpositions of DNMT1 with the nucleosome, where DNMT1-bound DNA is mapped to (**A**) DNA position 3 of the unpacked nucleosome complex, (**B**) DNA position 8 of the unpacked nucleosome complex, (**D**) DNA position 61 of the packed nucleosome complex, and (**E**) DNA position 64 of the packed nucleosome complex. If a DNA binding position is accessible to DNMT1 (i.e. the steric clash is below the critical threshold), the DNA position can be assumed as methylated by DNMT1. In (**C**) for the unpacked conformation and in (**F**) for the packed conformation, the y-axis represents the fraction of DNMT1 atoms that sterically overlap with any atom of the nucleosome across different DNA–nucleosome mapping positions, as displayed on the x-axis. In arrangements 3 and 64 of the unpacked and packed conformations, respectively, the DNA can be methylated. In contrast, in arrangements 8 and 61 of the unpacked and packed conformations, the DNA cannot be methylated (**C**, **F**). The resulting wave pattern of steric clashes reflects the 10 bp periodicity of double-stranded DNA. The scores for orientations at positions 3, 8, 61, and 64, shown in (**A**, **B**, **D**, and **E**), respectively, are highlighted by the red circles above. The vertical dashed lines in (**C** and **F**) separate the first and second DNA turns around the nucleosome

Methylation site classification provides insight into DNA accessibility to proteins: GpC methylation of specific sites reveals areas not protected by nucleosomes or tight-binding proteins [24], while CpG methylation indicates accessibility to DNA methyltransferase. A simple structural *in silico* approach was implemented such that the X-ray crystallographic structures of a DNMT1-DNA complex were superimposed onto every possible position of DNA wrapped around a histone octamer. Figures 6A and B illustrate two examples of these superimpositions of DNMT for an unpacked DNA-nucleosome complex. In the arrangement shown in Fig. 6A, DNMT1 and the nucleosome exhibit only minor atomic steric overlaps, which could readily be resolved through slight conformational adjustments of the proteins. In contrast, the hypothetical arrangement in Fig. 6B shows significant overlap, such that DNMT1 and the nucleosome "sit" atop each other and generate significant steric effects, rendering this arrangement impossible. Similarly, Figs. 6D and E illustrate two examples of DNMT1 superimposed on the packed DNA-nucleosome complex, with a high overlap and low overlap, respectively. For each aligned nucleosome-DNA position, the extent to which the three-dimensional structure of DNMT1 sterically overlaps with that of the nucleosome was assessed. Figures 6C and F depict the calculated degree of steric clash (%) as an inverse measure of DNA accessibility for each histone octamer-bound DNA position, for the unpacked and packed conformation, respectively. The resulting wave pattern reflects the periodic wrapping of DNA around the nucleosome, closely resembling the aforementioned 10 bp periodicity of CpG methylation levels.

### Correlating methylation levels and structural accessibility model

To investigate the statistical significance between accessible histone-bound regions and methylated CpG sites, an analysis for both nucleosome conformation models was conducted, examining expressed and non-expressed genes separately, by computing a match-score for every possible sliding window within the regions of interest for both HNDRs and LNDRs. After doing so for both experimental and randomized CpG methylation data, the match scores were compared using *p*-values derived from a Wilcoxon rank-sum test and effect size, computed by Cohen’s d. Given that the number of CpGs within a sliding window significantly impacts the reliability of the match score, both the effect size and statistical significance were examined as a function of the number of CpGs.

**Fig. 7:**
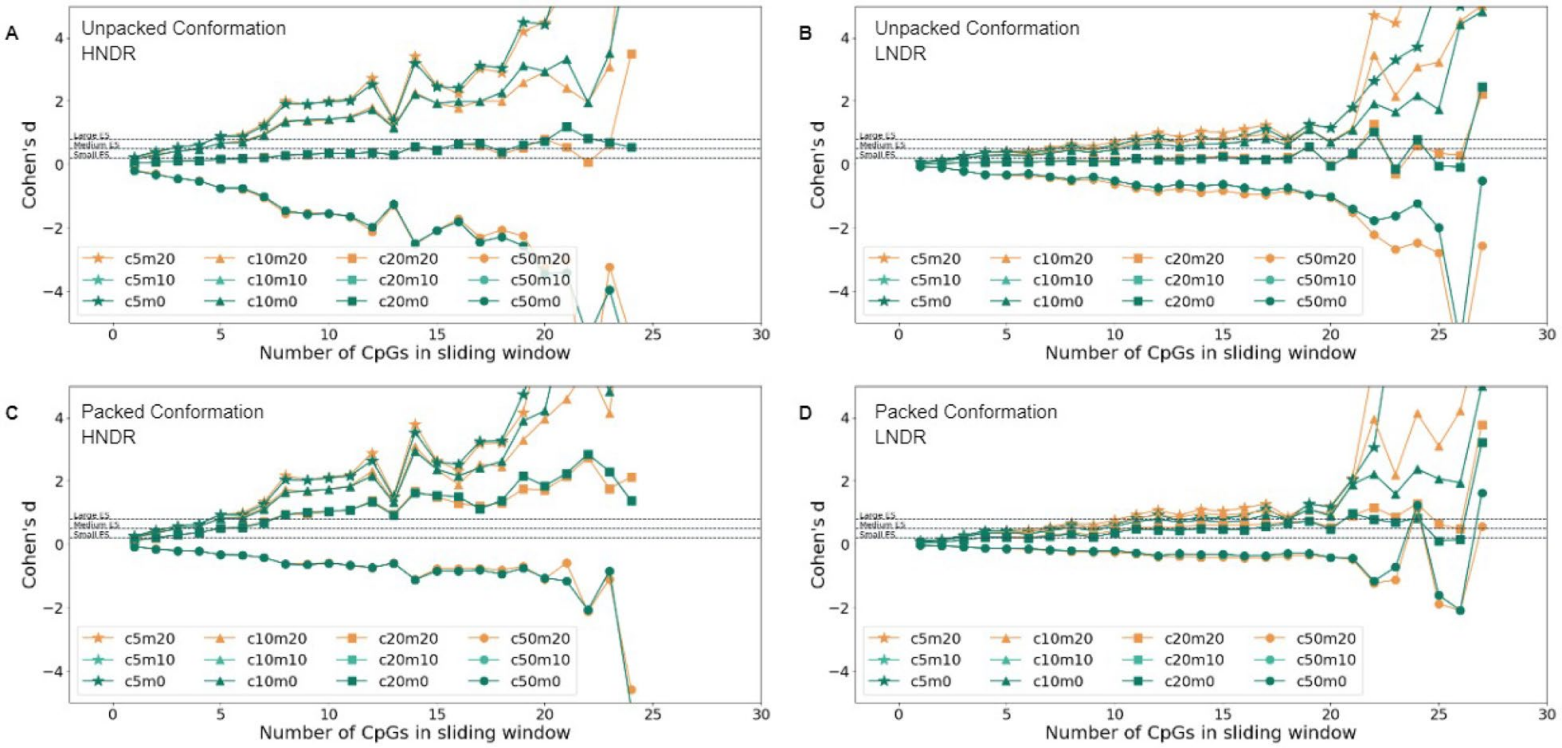
For 7xib.pdb, matching between methylation data and accessibility scores for HNDR and LNDR regions in expressed promoters. The top two panels show Cohen’s d values, with respect to the number of CpGs in the sliding window, for the packed state in (**A**) HNDR regions and (**B**) LNDR regions. Similarly, the bottom two panels show the same analysis for the unpacked state in (**C**) HNDR regions and (**D**) LNDR regions

The effect size for experimental and randomized data within HNDRs and LNDRs can be plotted as a function of the number of CpGs within a sliding window across the two nucleosomal packing conditions, as shown for the promoters of expressed genes in Fig. 7. Similar behaviour is observed in other regions; see Supplementary Figures S3-S10. LNDRs are used as a negative control, as it is assumed that these DNA regions are unlikely to be bound by nucleosomes, making all positions equally accessible to DNMTs. Abiding by this hypothesis, a greater effect in HNDRs is expected to be observed in comparison to LNDRs. Cohen’s d values and *p*-values were calculated across the following ranges: $m_{thres} \in [0,10,20]$ and $c_{thres} \in [5,10,20,50]$. Indeed, Cohen’s d values for promoters of expressed genes were clearly more pronounced in HNDR regions (Figs. 7A and C) than in LNDR regions (Figs. 7B and D). Furthermore, stronger effect sizes were detected when HNDR data was mapped to the packed chromatin conformation (Fig. 7C) than for the unpacked chromatin conformation (Fig. 7A). For comparison, Cohen’s d effect sizes were notably lower in the regions of interest for non-expressed genes; see Figures S5-S6 and S9-S10. There, results for HNDR regions were of comparable magnitude to those of LNDR regions. This suggests that neither the unpacked nor packed nucleosome structure fully reflects a representative structural model for genomic regions involving non-expressed genes.

It is observed that Cohen’s d values are highest when $c_{thres}=5$ for expressed genes in both packing conditions, indicating that a CpG position can be methylated if the steric clash between DNMT1 and the nucleosome is below 5%. As the threshold value $c_{thres}$ increases, the effect size decreases; particularly, at high clash values, the effect size can become negative, counterintuitively implying that the matching scores of randomized methylation data were, on average, higher than those of experimental methylation levels. Additionally, the impact of the parameter $m_{thres} \in [0,10,20]$ on the effect size was less significant than that of $c_{thres}$. Notably, the effect becomes more pronounced when 10 or more CpGs are present in the sliding window. Beyond $c_{thres}=20$, the quantity of datapoints quickly decreases, signifying that Cohen’s d values cannot be reliably computed in this regime; see rightmost panels of Fig. S3-S10. As a result, the Cohen’s d values were calculated for $c_{thres}=5$ and $m_{thresh}=0$ for sliding windows containing 10–20 CpGs, as shown in Table 1.

**Table 1:** Cohen’s d values for HNDRs and LNDRs for $c_{thres}=5$ and $m_{thres}=0$ for sliding windows containing 10 to 20 CpGs, based on accessibilities from 7XIB.pdb

Regions	Unpacked conformation		Packed conformation	
	HNDRs	LNDRs	HNDRs	LNDRs
Promoter	2.06	0.73	2.18	0.77
Start of 1$^{st}$ intron	3.26	1.22	3.47	1.28
End of 1$^{st}$ intron	2.30	1.72	2.35	1.78
Start of 2$^{nd}$ intron	2.77	1.66	2.84	1.72

As reflected in Table 1, HNDRs consistently exhibit a stronger effect compared to LNDRs in expressed genes, signifying that in HNDRs of actively transcribed regions, where nucleosomes are present, the DNA methylation pattern aligns with the structural accessibility of DNMT1 around a nucleosome. This difference of effect size across conformations is greatest at the promoter and at the start of the 1^st^ intron, indicating higher concurrence of methylation and DNMT1 accessibility in these regions. This effect is more pronounced in the packed nucleosome complex, suggesting that methylation in HNDRs aligns more closely with the structural accessibility of the packed conformation. In contrast, in LNDR regions for expressed genes, where nucleosomes are assumed to be absent, DNA is more accessible to DNMT1, implying that the DNA methylation pattern does not necessarily correspond to the structural accessibility of DNMT1 around a nucleosome, as reflected by the low Cohen’s d values.

In the regions near the end of the 1^st^ intron and the start of the 2^nd^ intron, łarger effect is observed in both LNDR and HNDR regions; this is likely attributed to the finding that, unlike promoters and the start of the 1^st^ intron whereby some nucleosome patterns are evident, no such patterns are observed (see Figs. 4C and D). In these regions, the average GpC methylation is consistently low, indicating a lack of LNDRs. Additionally, the detected LNDRs are dispersed across these regions (Figs. 4G and H) and do not show pronounced peaks near exon-intron boundaries, with respect to the start of the 1^st^ intron and the TSS. For non-expressed genes with parameters $c_{thres}=5$ and $m_{thres}=0$ (Figures S1 and S2), slightly higher effect sizes were derived in the packed conformation when compared to the unpacked conformation; larger effect size is shown for HNDRs in promoters and the start of the 1^st^ intron, while larger effect for LNDRs is observed at the end of the 1^st^ intron and at the start of the 2^nd^ intron.

Figure S11, as well as Table S3, show significant *p*-values and Cohen’s d values obtained with PDB structure 3PTA, which features a DNMT1 enzyme bound to a DNA stretch with non-flipped out bases. The trends of these results are highly similar to those obtained using PDB structure 7XIB. Yet, the Cohen’s d values are somewhat smaller when derived from structure 3PTA.

#### Limitations and implications

Notably, our study possesses clear limitations in terms of the structural superposition approach used. One of the atomic models employed in this study, a telomeric trinucleosome, is a specialized structure, the relevance of which to general chromatin organization is uncertain. This structure was intended to be representative of a more tightly packed chromosomal state than that of the single-nucleosome structure. Furthermore, the structural superimposition approach simply placed two rigid molecular complexes atop each other. This technique does not allow for intramolecular conformational changes that are often termed induced fit effects. In future work, one may extend this workflow by relaxing superimposed conformations via additional molecular modeling techniques, such as energy minimization or molecular dynamics simulations.

Additionally, steric hindrance is but one factor influencing DNMT1 access to DNA. Steric effects manifested most strongly at the start of the first exon, suggesting high relevance of precise methylation marks at this promoter position, concurring with the current view of transcriptional regulation. The lower correlation observed between nucleosome positioning and DNA methylation at the end of the first intron and at the start of the second intron may reflect that nucleosome positioning is not as precise in these regions, which is also in accordance with the current paradigm. Beyond DNA accessibility, DNMT1 activity is likely modulated by recruitment via specific histone marks, such as H4K20me3 [41] and H3K9me3 [42], via H3Ub2 [43], and by further processes, including dynamic nucleosome sliding, DNMT1 conformational plasticity, chromatin remodeling, histone exchange, and the action of histone chaperones. These processes may collectively modulate steric effects on DNA methylation and influence transient DNA accessibility, potentially differing across genomic regions.

One may question how low GpC methylation levels may impact Cohen’s d: if GpC methylation levels are consistently low, distinguishing between HNDR and LNDR regions becomes unclear. Our approach focuses on the analysis of CpG levels in HNDR regions; if HNDR regions are “diluted” by additional false-positive LNDR regions, this would limit the ability of our approach to detect nucleosome phasing patterns and effects of the 10-bp periodicity of DNA, such as the preferred CpG methylation in outward-facing positions, resulting in less significant Cohen’s d values. In LNDR regions, our analysis still identified statistically significant results in the same direction as HNDR regions, however with smaller Cohen’s d values; see Supplement. Hence, it is believed that analyzing data with low GpC levels will not lead to erroneous detection of false effects, but would rather lower the significance of existing methylation patterns.

In principle, the activity of DNMT1 is tightly linked to the cell cycle. If there exists a timepoint during the cell cycle at which DNMT1 could freely methylate DNA unbound from nucleosomes, it appears unlikely as to why this would result in a pronounced 10-bp phasing pattern at the promoter and start of the 1^st^ intron. Caron et al. [44] studied proliferation of human B cells into plasma cells and determined that this proliferation was linked to a slight decrease in DNA methylation levels, followed by a committal step in which an S phase-synchronized differentiation switch was associated with extensive DNA demethylation, as well as local acquisition of 5-hydroxymethylcytosine at enhancers and plasma cell-specific genes. Only marginal effect was attributed to cell cycle shifts, with stronger DNA methylation changes upon differentiation. Vandiver et al. [45] analyzed dermal fibroblasts in the G0, G1, and G2 phases and detected no global changes or large-scale hypomethylated blocks in any of the examined cell cycle phases. These findings argue against the relevance of cell-cycle dependent effects on the detected phasing effects of DNA methylation.

Comparing the data to the current understanding of chromatin structure, it has been observed in both *H. sapiens* and *A. thaliana* that nucleosomal DNA displays a 10-bp periodicity of methylated CpG sites, validating the periodicity observed in Figs. 6C and F. [22, 23, 46] Notably, some region-specific methylation patterns are implied in exon-intron boundaries. [47] The observed deficit in LNDRs near exon-intron boundaries matches findings indicating that exons possess higher degrees of methylation than introns, as well as increased nucleosome occupancy. [22, 23, 48, 49] Within an expressed gene, methylation "context" is needed: methylation at promoters impedes transcription and is implied in long-term silencing, whereas methylation within the gene body may elongate transcription and influence splicing. [50] Thus, in expressed regions, lower GpC and CpG methylation at the promoter and a greater presence of LNDRs before the TSS facilitate active transcription. Moreover, observed methylation patterns downstream of the promoter may play a regulatory role. Upon transcription, RNA Polymerase II preferentially binds to exons; nucleosome positioning at exon-intron boundaries may regulate RNA Polymerase II efficiency, enhancing splicing accuracy of upstream introns and mitigating exon skipping. [22] Differential DNA methylation within genes may drive exon definition and alternative promoter usage. [51]

Determining whether the observed correlations between accessibility and methylation are cell-type-specific or conserved across tissues or species, Salhab et al. [52] compared the DNA methylation landscape of primary human hepatocytes (PHH) to liver cancer tissue and hepatocellular carcinoma cell lines (HepaRG and HepG2). Notably, the methylome of primary liver cancer retained a pattern of partially methylated domains (PMDs) highly similar to primary cells. PMDs in cancer tissue display a mild, yet clearly reduced level of methylation. However, in cancer cell lines, the DNA methylation in PHH-specific PMDs is strongly decreased. In particular, the most significant changes were observed in gene-poor regions, whereas in gene-rich regions, DNA methylation and expression levels remained correlated. It is plausible that the nucleosome phasing pattern identified at promoters and at the start of the 1^st^ intron are quite general in human cell types. The connection between DNA accessibility and methylation patterns should be generalizable for human cell types, and possibly for other mammalian species.

Moreover, the workflow introduced in this study can potentially be used in combination with other methodologies (e.g. ATAC-Seq), as well as with data on other DNA-binding proteins, such as pioneer transcription factors.

## Conclusion

Here, a simple computational scheme was introduced to quantify the relationship between the structural accessibility of nucleosome-bound DNA to DNMT1 and observed genome-wide DNA methylation patterns in human HepG2 cells. It has been previously suggested that DNA methylation and nucleosome occupancy are dependent on each other, perhaps in a bidirectional manner [22, 36, 53]. Chodavarapu et al. suggested that DNA nucleotides pointing away from the nucleosome are more accessible to DNA methyltransferases, yielding methylation periodicity [22]. The results obtained from our structural analysis support this hypothesis and suggest that while nucleosome–bound DNA can indeed be methylated by DNMT1, sterically accessible CpG sites are methylated to a larger extent, on average. Statistically, for expressed genes, it is shown that methylation patterns align most significantly in packed nucleosomal arrays; however, for non-expressed genes, no such consistent correlation emerges and it is unclear which structural conformation reflects the biological paradigm. Whether this indicates that DNMT1 is only active on nucleosome-bound DNA or if these positions are methylated prior to binding to the nucleosome is beyond the scope of this analysis. The aim of this study is to provide a mechanistic structural model for the hypothesis of Chodavarapu et al. and quantify its statistical significance. Nevertheless, several other factors, such as histone modifications, are reported to influence DNA methylation, as well [54]. For future directions, consideration of present and absent histone modifications, as well as more complex histone structure, may further improve the understanding of this process and aid in deciphering underlying methylation patterns.

## Supplementary information

Supplementary figures S1 and S2 show analogous GpC, LNDR, HNDR, and CpG data for non-expressed genes. Supplementary figures S3 to S10 display Cohen’s D values, statistical significance, and data coverage similar to Fig. 7 in the main text for all other entries listed in table 1. Supplementary tables S1 and S2 list Cohen’s d values for varying thresholding parameters.

## Additional file

Supplementary file 1.

## Data Availability

NoME-seq data was retrieved from the European Phenome Archive (EGAD00001002527). RNAseq data was retrieved from NCBI (GEO accession: GSE206417). Code for structural superimposition and steric plots are available at https://github.com/kevingeo11/dnaMethylPatterns
