# Supplementary material for: Structural DNMT-nucleosome contacts are related to DNA methylation patterns
Source: Epigenetics Chromatin. 2025 Sep 12;18:59. doi: 10.1186/s13072-025-00626-1 (PMC12427118; doi:10.1186/s13072-025-00626-1)
Supplement: Supplementary file 1 [file 13072_2025_626_MOESM1_ESM.pdf]

Supplementary Material: Structural DNMT-Nucleosome Contacts are Related to DNA Methylation patterns

Kevin George[1], Kerstin Neininger[1], Anna Elizabeth Schmitz [1], Jörn Walter[2], Volkhard Helms[1*]

[1]Center for Bioinformatics, Saarland University, Saarbrücken, Germany.
[2]Department of Genetics, Saarland University, Saarbrücken, Germany.

*Corresponding author(s). E-mail(s): volkhard.helms@bioinformatik.uni-saarland.de;
Contributing authors: kege00001@stud.uni-saarland.de; kerstin.neininger@gmail.com; ansc00053@uni-saarland.de; j.walter@mx.uni-saarland.de;

Supplementary Figures

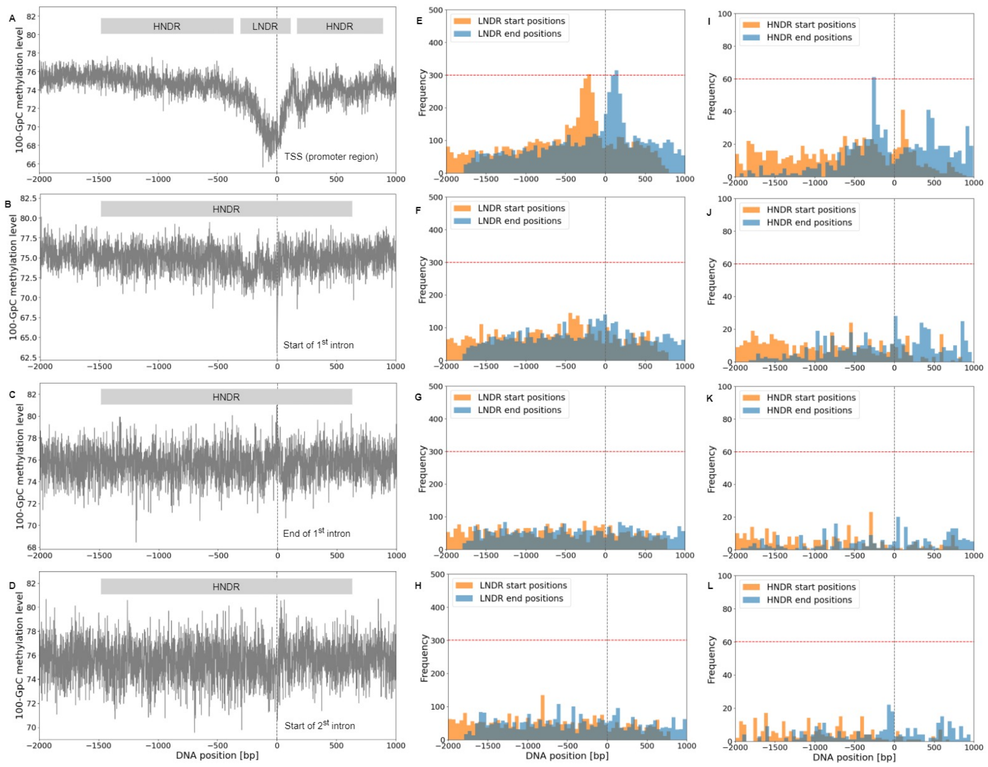

Fig. S1 NOMe–seq GpC patterns and start/end distributions of LNDRs and HNDRs for non-expressed genes. (A-D) Average 100–GpC methylation levels (percent of unmethylated GpCs) indicating nucleosome depleted and occupied regions for all four regions of interest. (E-L) Regions having higher nucleosome density than the local surrounding (HNDRs) and regions with lower nucleosome density (LNDRs) were derived based on experimental GCH NOMe–seq data. Shown are distributions of start/end positions of LNDRs (E-H) and HNDRs (I-L).

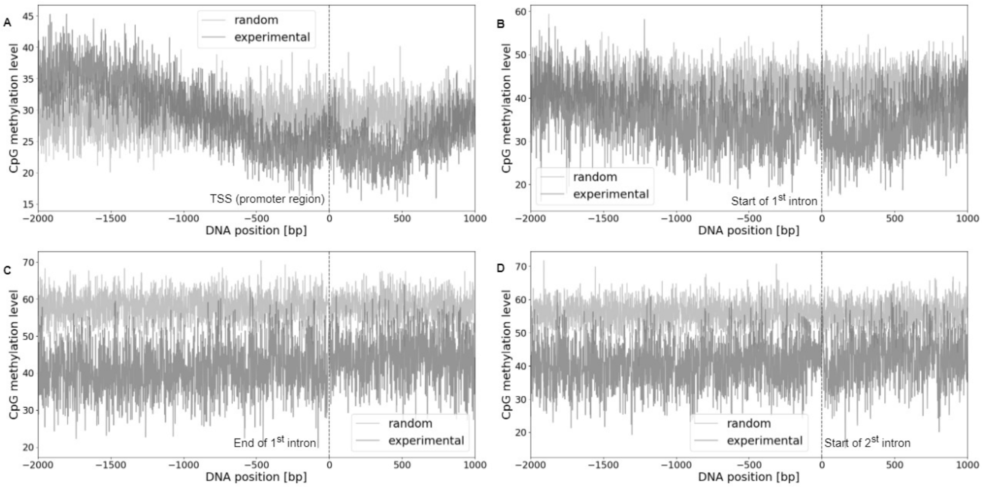

Fig. S2 CpG methylation pattern of experimental and randomized data for non expressed genes. Shown is the general CpG pattern for experimental (dark grey) and randomized (light grey) methylation levels in the 4 regions. We observe a distinct difference between the pattern of average experimental CpG methylation and that of randomized CpG methylation.

Table S1 Cohen's d values for HNDRs and LNDRs for sliding windows containing 10 to 20 CpGs across all thresholding parameters for expressed and non expressed genes for the unpacked conformation

		Expressed		Non-expressed	
Regions	Parameter	HNDRs	LNDRs	HNDRs	LNDRs
promoter	c5m0	0.97	0.38	0.54	0.41
	c5m10	0.97	0.38	0.54	0.41
	c5m20	0.97	0.45	0.61	0.45
	c10m0	0.52	0.21	0.31	0.23
	c10m10	0.52	0.21	0.31	0.22
	c10m20	0.52	0.24	0.35	0.25
	c20m0	-0.30	-0.12	-0.21	-0.14
	c20m10	-0.30	-0.12	-0.21	-0.13
	c20m20	-0.29	-0.15	-0.23	-0.14
	c50m0	-1.72	-0.64	-0.83	-0.70
	c50m10	-1.72	-0.64	-0.83	-0.69
	c50m20	-1.74	-0.76	-0.92	-0.75
start of 1st intron	c5m0	1.50	0.66	0.78	0.61
	c5m10	1.50	0.66	0.78	0.61
	c5m20	1.54	0.72	0.85	0.68
	c10m0	0.80	0.37	0.45	0.34
	c10m10	0.81	0.37	0.45	0.34
	c10m20	0.82	0.40	0.49	0.38
	c20m0	-0.45	-0.22	-0.28	-0.20
	c20m10	-0.45	-0.22	-0.28	-0.20
	c20m20	-0.46	-0.24	-0.31	-0.23
	c50m0	-2.66	-1.08	-1.14	-1.01
	c50m10	-2.67	-1.09	-1.14	-1.01
	c50m20	-2.72	-1.19	-1.28	-1.10
end of 1st intron	c5m0	1.36	1.00	0.79	1.14
	c5m10	1.35	1.00	0.78	1.14
	c5m20	1.38	1.04	0.80	1.19
	c10m0	0.78	0.56	0.44	0.61
	c10m10	0.77	0.56	0.43	0.61
	c10m20	0.80	0.59	0.45	0.64
	c20m0	-0.42	-0.32	-0.26	-0.32
	c20m10	-0.42	-0.32	-0.26	-0.32
	c20m20	-0.43	-0.34	-0.26	-0.35
	c50m0	-2.07	-1.52	-1.22	-1.89
	c50m10	-2.05	-1.51	-1.21	-1.89
	c50m20	-2.08	-1.57	-1.20	-1.97
start of 2nd intron	c5m0	1.44	0.95	0.85	1.11
	c5m10	1.44	0.95	0.85	1.11
	c5m20	1.43	1.01	0.92	1.22
	c10m0	0.77	0.53	0.51	0.60
	c10m10	0.77	0.54	0.51	0.60
	c10m20	0.77	0.57	0.54	0.66
	c20m0	-0.45	-0.29	-0.26	-0.33
	c20m10	-0.45	-0.29	-0.26	-0.33
	c20m20	-0.45	-0.32	-0.29	-0.37
	c50m0	-2.42	-1.47	-1.24	-1.81
	c50m10	-2.42	-1.47	-1.24	-1.81
	c50m20	-2.37	-1.55	-1.37	-2.00

Table S2 Cohen's d values for HNDRs and LNDRs for sliding windows containing 10 to 20 CpGs across all thresholding parameters for expressed and non expressed genes for the packed conformation

		Expressed		Non-expressed	
Regions	Parameter	HNDRs	LNDRs	HNDRs	LNDRs
promoter	c5m0	1.63	0.61	0.80	0.66
	c5m10	1.63	0.61	0.80	0.66
	c5m20	1.64	0.72	0.89	0.72
	c10m0	1.26	0.48	0.68	0.52
	c10m10	1.26	0.48	0.68	0.52
	c10m20	1.25	0.57	0.76	0.56
	c20m0	0.58	0.22	0.33	0.24
	c20m10	0.58	0.22	0.33	0.24
	c20m20	0.58	0.26	0.38	0.26
	c50m0	-0.53	-0.23	-0.35	-0.25
	c50m10	-0.53	-0.23	-0.35	-0.25
	c50m20	-0.52	-0.27	-0.38	-0.28
start of 1st intron	c5m0	2.49	1.03	1.11	0.96
	c5m10	2.49	1.03	1.11	0.96
	c5m20	2.54	1.13	1.24	1.06
	c10m0	1.87	0.82	0.95	0.77
	c10m10	1.87	0.83	0.95	0.77
	c10m20	1.90	0.90	1.06	0.86
	c20m0	0.85	0.39	0.50	0.36
	c20m10	0.85	0.39	0.50	0.36
	c20m20	0.86	0.43	0.55	0.41
	c50m0	-0.84	-0.42	-0.48	-0.39
	c50m10	-0.83	-0.42	-0.48	-0.39
	c50m20	-0.85	-0.46	-0.53	-0.42
end of 1st intron	c5m0	2.01	1.48	1.20	1.83
	c5m10	2.00	1.47	1.18	1.83
	c5m20	2.02	1.53	1.18	1.90
	c10m0	1.65	1.21	1.03	1.41
	c10m10	1.64	1.21	1.01	1.41
	c10m20	1.66	1.27	1.02	1.45
	c20m0	0.83	0.61	0.54	0.68
	c20m10	0.82	0.61	0.54	0.68
	c20m20	0.84	0.65	0.54	0.68
	c50m0	-0.78	-0.55	-0.49	-0.64
	c50m10	-0.78	-0.56	-0.49	-0.64
	c50m20	-0.81	-0.59	-0.49	-0.69
start of 2nd intron	c5m0	2.31	1.42	1.23	1.73
	c5m10	2.31	1.42	1.23	1.73
	c5m20	2.26	1.50	1.35	1.91
	c10m0	1.80	1.16	1.06	1.37
	c10m10	1.80	1.16	1.06	1.37
	c10m20	1.76	1.23	1.16	1.50
	c20m0	0.84	0.58	0.59	0.67
	c20m10	0.84	0.58	0.59	0.67
	c20m20	0.83	0.62	0.62	0.72
	c50m0	-0.83	-0.55	-0.51	-0.65
	c50m10	-0.83	-0.55	-0.51	-0.65
	c50m20	-0.85	-0.59	-0.55	-0.71

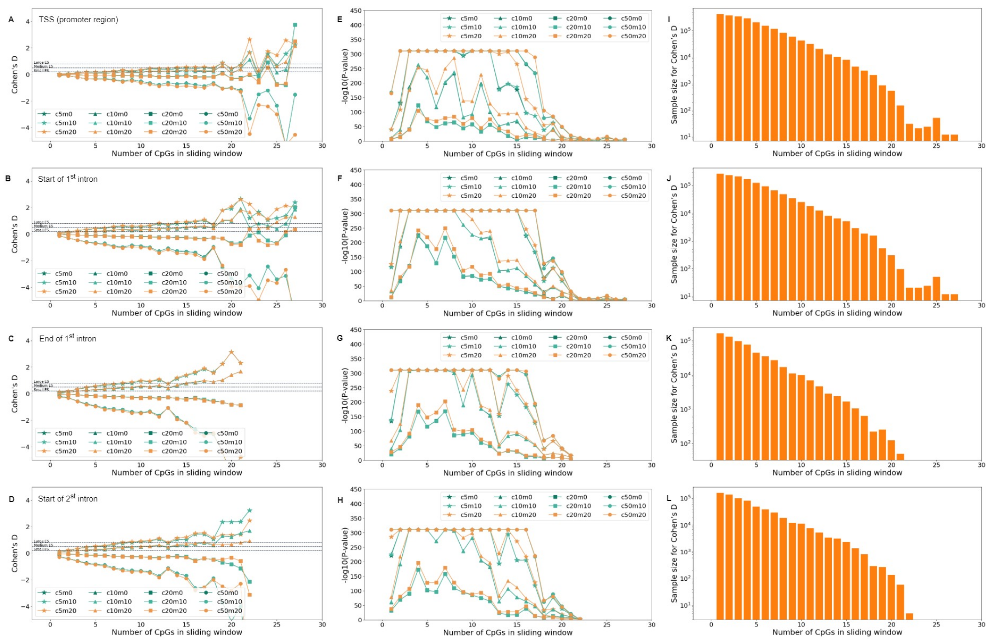

Fig. S3 Matching between methylation data and accessibility scores of the unpacked conformation for LNDR regions for expressed genes. (A-D) Cohen's d values with respect to the number of CpGs in the sliding window for (A) promoters, (B) start of 1st intron, (C) end of 1st intron and (D) start of 2nd intron. (E-H) p–values between experimental and randomly shuffled data calculated with the Wilcoxon rank–sum test for (E) promoters, (F) start of 1st intron, (G) end of 1st intron and (H) start of 2nd intron. For numerical reasons, $-log_{10}(p - value) = 310$ is the maximum and thus the smallest p-value possible. (I-L) The sample size for methylation data as a function of the number of CpGs for (I) promoters, (J) start of 1st intron, (K) end of 1st intron and (L) start of 2nd intron.

Table S3 For structure 3pta, Cohen's d values for HNDRs and LNDRs for $c_{thres} = 5$ and $m_{thres} = 0$ for sliding windows containing 10 to 20 CpGs.

Regions	Unpacked Conformation		Packed Conformation	
	HNDRs	LNDRs	HNDRs	LNDRs
promoter	0.97	0.38	1.63	0.61
start of 1st intron	1.50	0.66	2.49	1.03
end of 1st intron	1.36	1.00	2.01	1.48
start of 2nd intron	1.44	0.95	2.31	1.42

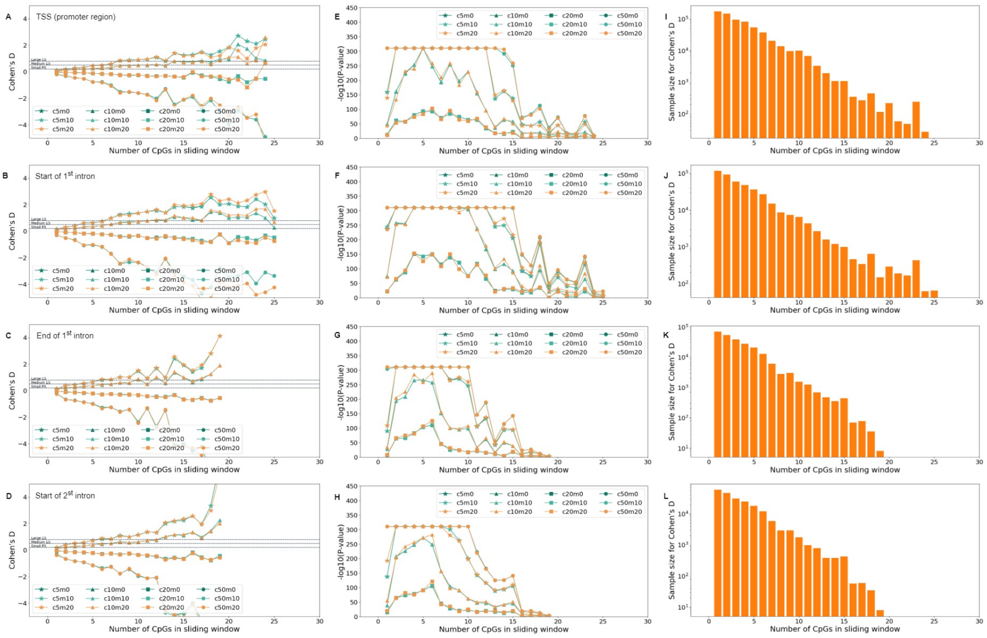

Fig. S4 Matching between methylation data and accessibility scores of the unpacked conformation for HNDR regions for expressed genes. (A-D) Cohen's d values with respect to the number of CpGs in the sliding window for (A) promoters, (B) start of 1st intron, (C) end of 1st intron and (D) start of 2nd intron. (E-H) p–values between experimental and randomly shuffled data calculated with the Wilcoxon rank–sum test for (E) promoters, (F) start of 1st intron, (G) end of 1st intron and (H) start of 2nd intron. For numerical reasons, $-log_{10}(p - value) = 310$ is the maximum and thus the smallest p-value possible. (I-L) The sample size for methylation data as a function of the number of CpGs for (I) promoters, (J) start of 1st intron, (K) end of 1st intron and (L) start of 2nd intron.

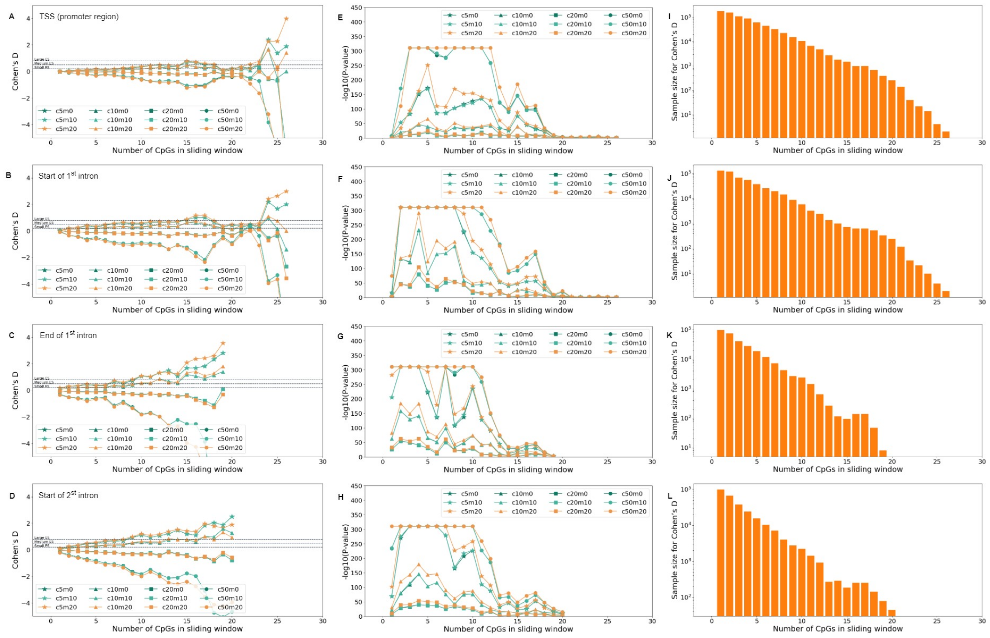

Fig. S5 Matching between methylation data and accessibility scores of the unpacked conformation for LNDR regions for non expressed genes. (A-D) Cohen's d values with respect to the number of CpGs in the sliding window for (A) promoters, (B) start of 1st intron, (C) end of 1st intron and (D) start of 2nd intron. (E-H) p–values between experimental and randomly shuffled data calculated with the Wilcoxon rank–sum test for (E) promoters, (F) start of 1st intron, (G) end of 1st intron and (H) start of 2nd intron. For numerical reasons, $-log_{10}(p - value) = 310$ is the maximum and thus the smallest p-value possible. (I-L) The sample size for methylation data as a function of the number of CpGs for (I) promoters, (J) start of 1st intron, (K) end of 1st intron and (L) start of 2nd intron.

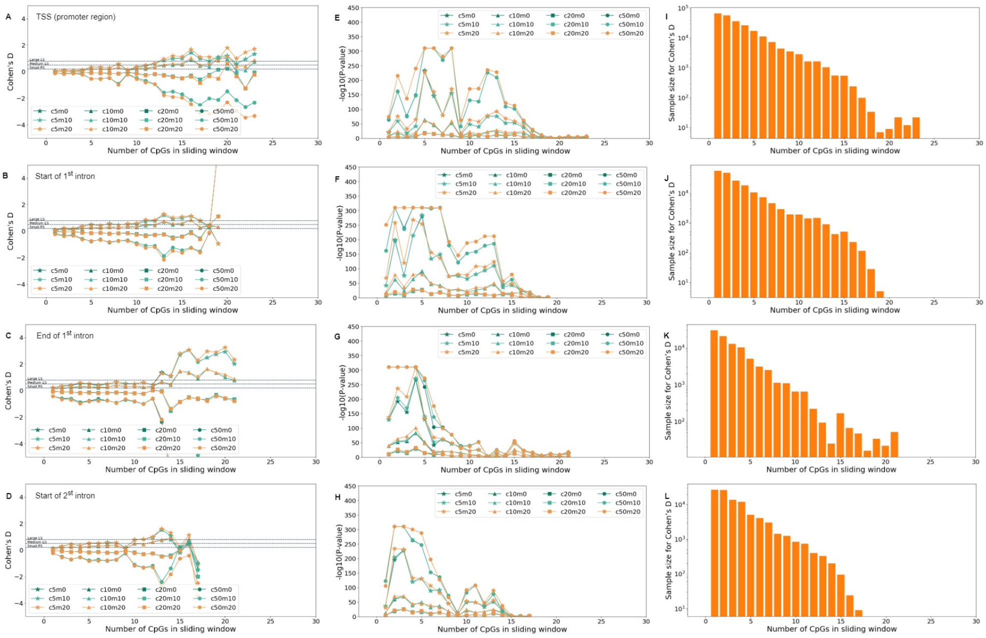

Fig. S6 Matching between methylation data and accessibility scores of the unpacked conformation for HNDR regions for non expressed genes. (A-D) Cohen's d values with respect to the number of CpGs in the sliding window for (A) promoters, (B) start of 1st intron, (C) end of 1st intron and (D) start of 2nd intron. (E-H) p–values between experimental and randomly shuffled data calculated with the Wilcoxon rank–sum test for (E) promoters, (F) start of 1st intron, (G) end of 1st intron and (H) start of 2nd intron. For numerical reasons, $-log_{10}(p - value) = 310$ is the maximum and thus the smallest p-value possible. (I-L) The sample size for methylation data as a function of the number of CpGs for (I) promoters, (J) start of 1st intron, (K) end of 1st intron and (L) start of 2nd intron.

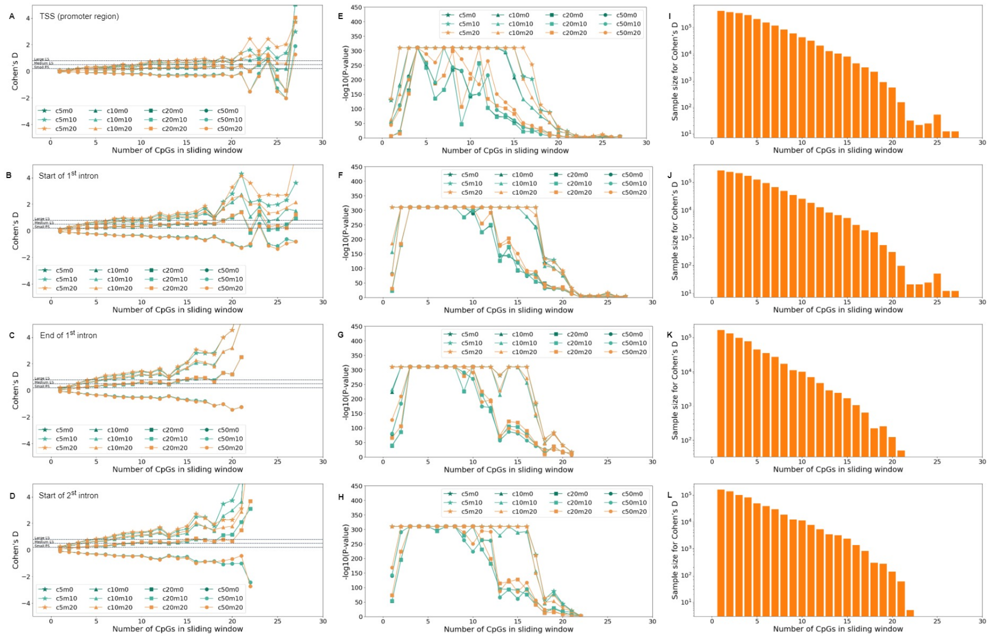

Fig. S7 Matching between methylation data and accessibility scores of the packed conformation for LNDR regions for expressed genes. (A-D) Cohen's d values with respect to the number of CpGs in the sliding window for (A) promoters, (B) start of 1st intron, (C) end of 1st intron and (D) start of 2nd intron. (E-H) p–values between experimental and randomly shuffled data calculated with the Wilcoxon rank–sum test for (E) promoters, (F) start of 1st intron, (G) end of 1st intron and (H) start of 2nd intron. For numerical reasons, $-log_{10}(p-value) = 310$ is the maximum and thus the smallest p-value possible. (I-L) The sample size for methylation data as a function of the number of CpGs for (I) promoters, (J) start of 1st intron, (K) end of 1st intron and (L) start of 2nd intron.

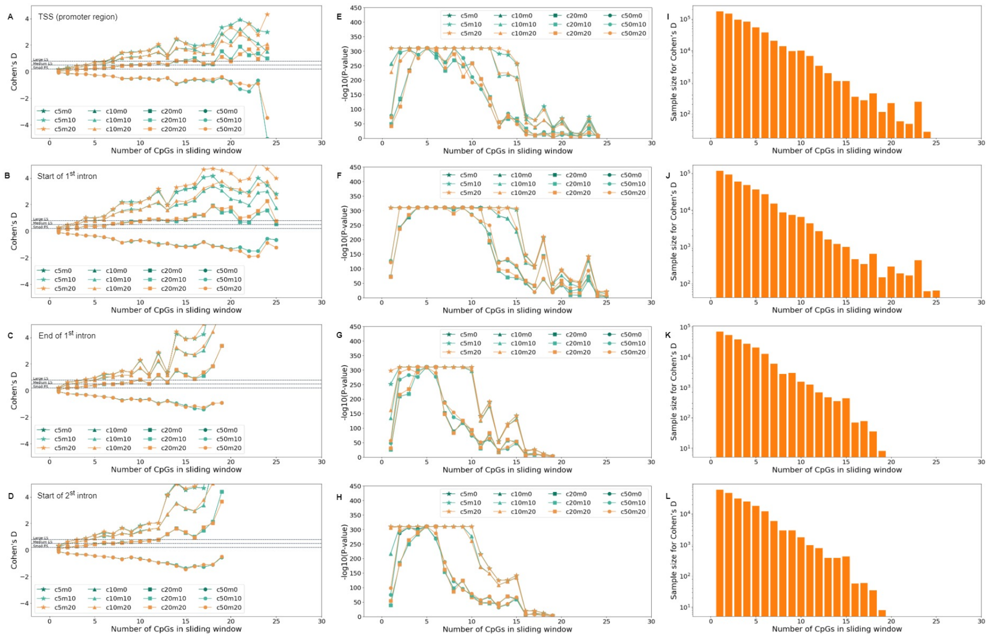

Fig. S8 Matching between methylation data and accessibility scores of the packed conformation for HNDR regions for expressed genes. (A-D) Cohen's d values with respect to the number of CpGs in the sliding window for (A) promoters, (B) start of 1st intron, (C) end of 1st intron and (D) start of 2nd intron. (E-H) p–values between experimental and randomly shuffled data calculated with the Wilcoxon rank–sum test for (E) promoters, (F) start of 1st intron, (G) end of 1st intron and (H) start of 2nd intron. For numerical reasons, $-log_{10}(p-value) = 310$ is the maximum and thus the smallest p-value possible. (I-L) The sample size for methylation data as a function of the number of CpGs for (I) promoters, (J) start of 1st intron, (K) end of 1st intron and (L) start of 2nd intron.

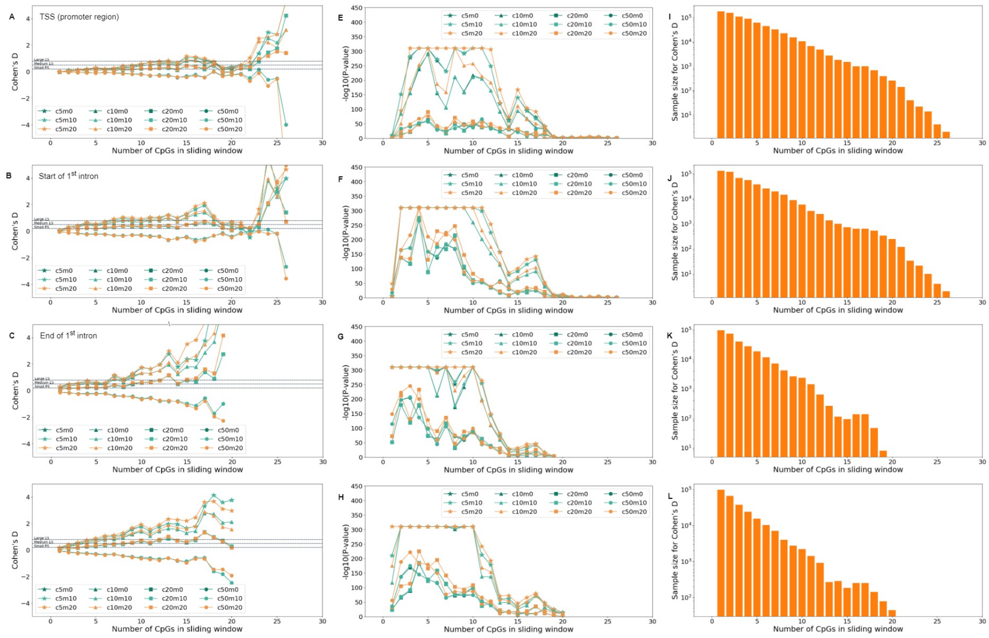

Fig. S9 Matching between methylation data and accessibility scores of the packed conformation for LNDR regions for non expressed genes. (A-D) Cohen's d values with respect to the number of CpGs in the sliding window for (A) promoters, (B) start of 1st intron, (C) end of 1st intron and (D) start of 2nd intron. (E-H) p–values between experimental and randomly shuffled data calculated with the Wilcoxon rank–sum test for (E) promoters, (F) start of 1st intron, (G) end of 1st intron and (H) start of 2nd intron. For numerical reasons, $-log_{10}(p - value) = 310$ is the maximum and thus the smallest p-value possible. (I-L) The sample size for methylation data as a function of the number of CpGs for (I) promoters, (J) start of 1st intron, (K) end of 1st intron and (L) start of 2nd intron.

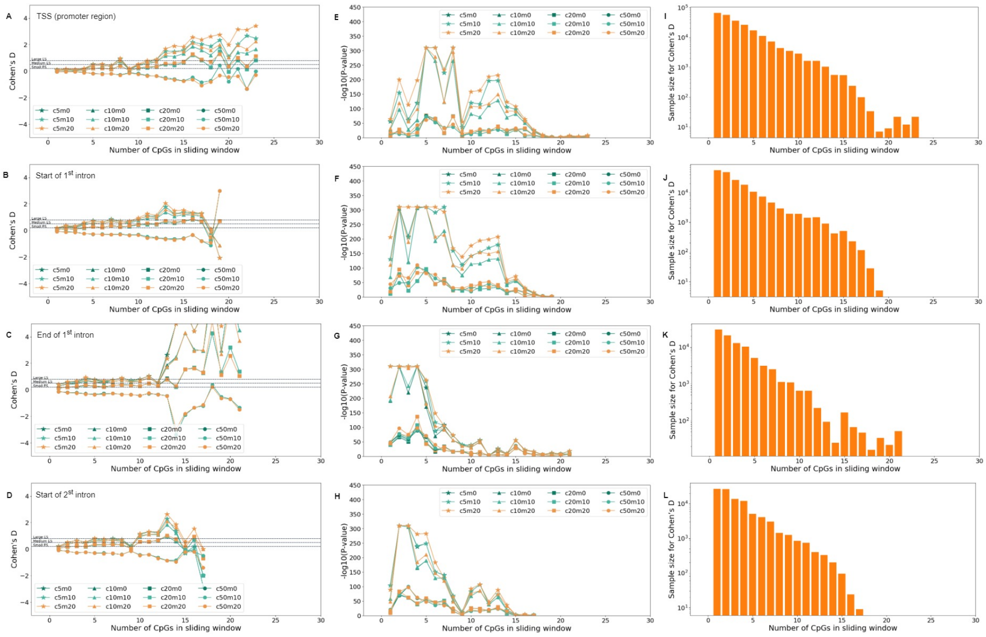

Fig. S10 Matching between methylation data and accessibility scores of the packed conformation for HNDR regions for non expressed genes. (A-D) Cohen's d values with respect to the number of CpGs in the sliding window for (A) promoters, (B) start of 1st intron, (C) end of 1st intron and (D) start of 2nd intron. (E-H) p–values between experimental and randomly shuffled data calculated with the Wilcoxon rank–sum test for (E) promoters, (F) start of 1st intron, (G) end of 1st intron and (H) start of 2nd intron. For numerical reasons, $-log_{10}(p - value) = 310$ is the maximum and thus the smallest p-value possible. (I-L) The sample size for methylation data as a function of the number of CpGs for (I) promoters, (J) start of 1st intron, (K) end of 1st intron and (L) start of 2nd intron.

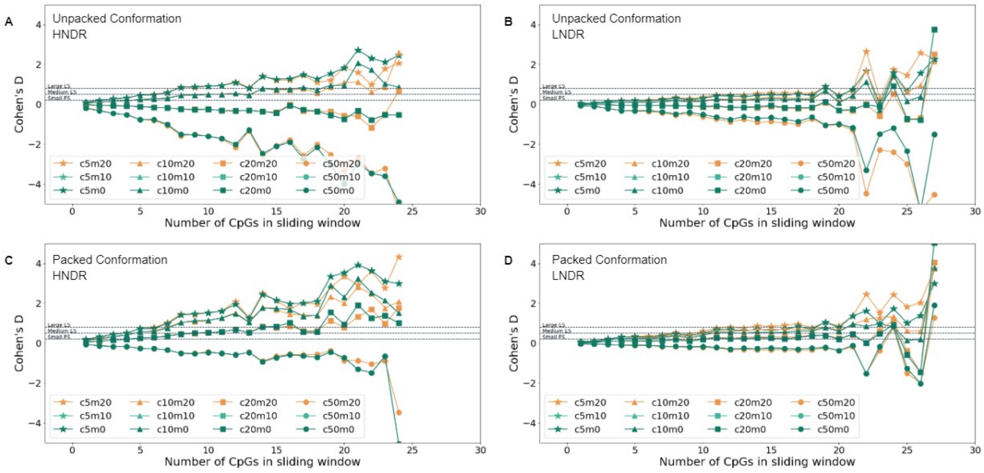

Fig. S11 For structure 3pta, matching between methylation data and accessibility scores for HNDR and LNDR regions in expressed promoters. The top panels show Cohen's d values, with respect to the number of CpGs in the sliding window, for the packed state in (A) HNDR regions and (B) LNDR regions. Similarly, the bottom panels show the same analysis for the unpacked state in (C) HNDR regions and (D) LNDR regions.
